# A Mobile Application Intervention for Emotional Health in Older Adults: Designed for Dementia Prevention

**DOI:** 10.3390/healthcare14162451

**Published:** 2026-08-07

**Authors:** Sang Ho Han, Se Hee Kong

**Affiliations:** 1 Department of Orthopedic Surgery, Daechan Hospital, 590 Inju-daero, Namdong-gu, Incheon 21570, Republic of Korea; 2 Hospital Daechan Sports Medical Research Center, 590 Inju-daero, Namdong-gu, Incheon 21570, Republic of Korea

**Keywords:** dementia prevention, mobile applications, aged, depression, life satisfaction

## Abstract

**Highlights:**

**What are the main findings?**

- A 7-month mobile application-based dementia prevention program was associated with a significant reduction in depressive symptoms among community-dwelling older adults.
- After adjustment for baseline differences, the intervention group showed significantly better post-intervention anxiety outcomes, whereas no significant between-group change was observed for life satisfaction.

**What are the implications of the main findings?**

- Multidomain mobile health interventions may support not only dementia prevention efforts but also emotional well-being in older adults living in the community.
- Application-based programs such as MELI may offer a practical, scalable, and accessible non-pharmacological strategy for community-based healthy aging initiatives.

**Abstract:**

**Background/Objectives:** With increasing aging in the global population, dementia prevalence continues to rise. Mobile health (mHealth)-based cognitive interventions have emerged as widely used non-pharmacological approaches for dementia prevention. In this study, we aimed to evaluate how the dementia-prevention application MELI affects depression, anxiety, and life satisfaction in older adults. **Methods:** Participants were community-dwelling older adults aged ≥ 60 years without a prior diagnosis of dementia. While the target sample size was 24 participants per arm based on an a priori power calculation using G Power, the achieved sample size reached 23 participants per arm (N = 46; experimental group = 23, control group = 23). The experimental group engaged in a 7-month intervention delivered through the MELI application, designed to support dementia prevention by providing cognitive training, physical activity guidance, and emotional support. Outcomes were assessed pre- and post-intervention using structured self-report questionnaires. Data were analyzed with repeated-measures analysis of covariance. **Results:** Compared to the control, depression reduced (*p* < 0.05, partial η^2^ = 0.227) and anxiety improved (*p* < 0.05, partial η^2^ = 0.110) statistically significantly in the experimental group. However, changes in life satisfaction did not reach statistical significance (*p* > 0.05, partial η^2^ = 0.000). **Conclusions:** The MELI mobile application might serve as a feasible digital intervention to support dementia prevention and promote emotional health in older adults. While depression and anxiety outcomes notably improved, further studies with larger samples would be required to clarify effects on life satisfaction. Mobile health-based programs represent a practical approach to dementia prevention in community-dwelling older adults.

## 1. Introduction

Owing to global population aging, the number of individuals living with dementia is expected to increase, with estimates suggesting approximately 150 million cases worldwide by 2050 [1,2]. Consequently, the social and economic burden of dementia is expected to escalate further, reaching approximately USD 2.8 trillion globally by 2030 [3,4]. These projections highlight dementia as a major global health challenge with far-reaching consequences for affected individuals and society. Accordingly, increased attention has been directed toward the development and implementation of preventive strategies.

Various non-pharmacological intervention strategies have been proposed to prevent dementia. Mobile application-based interventions are rapidly advancing because of progress in information technology, widespread smartphone use, and relative cost-effectiveness. The current literature suggests that dementia and cognition-related mobile applications can be categorized into cognitive screening and assessment applications, cognitive training-focused applications, emotional and quality-of-life support applications, and multidomain dementia prevention applications. Recently, cognitive screening applications utilizing digital biomarkers, such as eye tracking or machine learning techniques, have been proposed [5,6]. These studies focused on improving the accuracy of dementia screening and identifying cognitive decline at an early stage. However, a comprehensive review of these research trends suggests that despite their functional diversity, research on dementia prevention applications remains largely skewed toward cognition-centered technical innovation.

Livingston et al. reported that modifiable risk factors, such as low education level, hypertension, depression, obesity, and insufficient physical activity, are associated with an increased risk of dementia. These risk factors include emotional factors such as depression, underscoring the need to address psychological determinants alongside cognitive function in dementia prevention interventions. Therefore, interventions dealing with depression can be an important component in dementia-prevention strategies. Previous studies have reported that individuals with depression are at a greater risk of developing dementia than those without depression, regardless of when depressive symptoms first emerge [7,8]. From this perspective, rather than using a single-function approach, mobile applications aimed at dementia prevention should adopt an intervention model integrating cognitive, emotional, and lifestyle factors. Furthermore, mobile applications reportedly provide benefits such as reducing depression and stimulating cognitive function [9].

In this context, recent studies have not been limited to cognitive function improvement but have expanded their scope to care support or patient symptom management. For example, Salahub et al. verified effects of managing risk factors in the area and maintaining home care in the elderly including in those with dementia through a remote monitoring application [10], and Cho et al. presented results of a study evaluating the possibility of customized mobile application-based non-drug interventions for patients with dementia to alleviate behavioral and psychological symptoms (BPSD) [11]. However, these studies are mainly focused on care support or the symptom management of patients already diagnosed with dementia; studies that comprehensively evaluate emotional outcomes such as depression, anxiety, and life satisfaction from a preventive point of view are still insufficient. Therefore, a multidimensional approach would be required rather than a single function-oriented one, representing a need particularly noticeable in Korea, where the society is rapidly aging. Han et al. stated that Korea demonstrates an unprecedented pace of demographic change, reaching the transition from an aging society (defined as 7% of the population aged 65 and over) to a super-aged society (20%) in only 25 years (2000–2025), whereas other countries have taken between 50 and 100 years to undergo the same shift [12]. Against this backdrop, mobile applications reflecting the concept of multi-disciplinary dementia prevention are being developed in South Korea. For example, the MELI application (version 3.1) provides integrated content for older adults, including physical activity methods, cognitive training, and emotional well-being enhancement. Specifically, MELI offers structured exercise programs, puzzle-based and game-based cognitive training modules, mindfulness and meditation practices for emotional stability, and simple mental-health assessments, thereby delivering a comprehensive set of tools within a single platform. In addition, the application incorporates a user-friendly interface tailored to older adults, ensuring accessibility and sustained engagement.

However, research evaluating the effectiveness of multidisciplinary dementia prevention applications remain limited. Current application-based dementia prevention research has primarily focused on improving cognitive function or predicting dementia, whereas emotional factors such as depression and anxiety, as well as life satisfaction indicators, have been relatively neglected. Nevertheless, recent evidence from digital health interventions supports the potential relevance of such emotional factors. For instance, a systematic review and meta-analysis demonstrated that information technology-based cognitive behavioral therapy significantly reduced depression and anxiety symptoms among older adults [13].

In this study, the concept of intervention was clearly distinguished, and the scope of the study was established. Dementia prevention refers to a comprehensive approach to lowering the risk of development, and cognitive training refers to an activity that strengthens specific cognitive functions such as memory and attention. Emotional health support includes interventions that promote psychological stability and emotional well-being, such as depression, anxiety, and stress management; quality-of-life improvement aims to improve life satisfaction that encompasses physical, emotional, and social areas. Through this classification, the MELI application can be understood as a multi-functional dementia prevention tool that goes beyond simple cognitive training to emotional stability and improvement of life satisfaction.

This study aimed to explore the possibility that MELI intervention is associated with a reduction in depression and anxiety symptoms and an improvement in life satisfaction, and these results are expected to provide a basis for further research. The hypothesis of this study are as follows. First, the experimental group using the MELI application has the potential to reduce depression and anxiety symptoms and improve life satisfaction compared to the control group. Second, a multi-level approach that integrates physical activity and emotional well-being promotion is not limited to simple cognitive function improvement but is expected to provide psychological and emotional benefits.

## 2. Materials and Methods

### 2.1. Study Design

**Figure 1:**
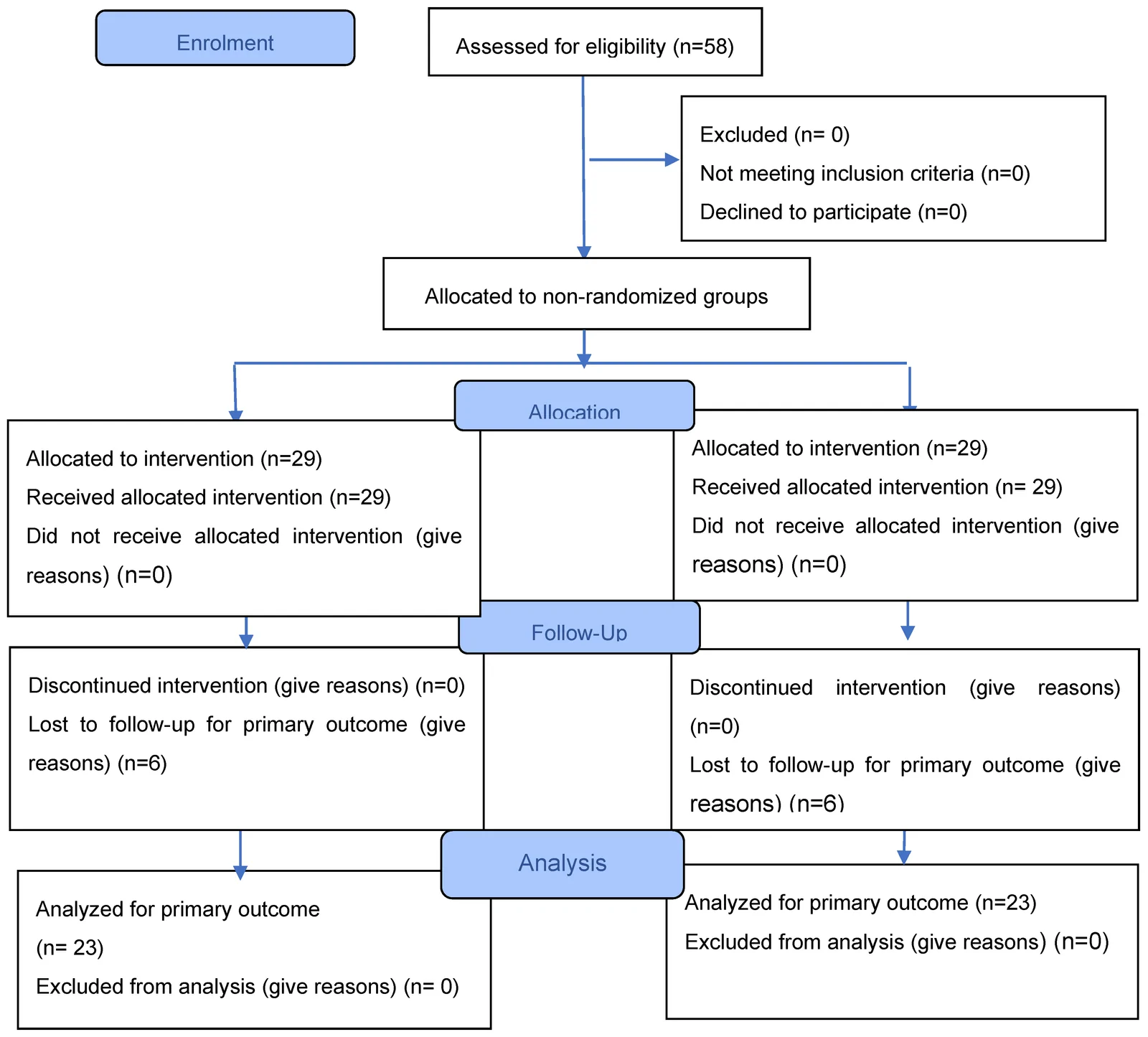
TREND flow diagram.

This quasi-experimental, two-group pretest–posttest study evaluated the emotional outcomes associated with the use of a mobile application for dementia prevention among community-dwelling older adults. Participants were allocated to the intervention or control group according to the type of intervention received. The intervention group used the dementia-prevention mobile application; the control group did not use the dementia-prevention application, and participants continued with their usual daily routines, which generally consisted of household tasks, light physical activity such as walking, and leisure activities including reading or watching television. Because no attention-control condition was implemented, the possibility of Hawthorne or expectancy effects should be considered when interpreting the findings. This study adheres to the TREND reporting guidelines for non-randomized designs, and the TREND flow diagram is provided in Figure 1.

### 2.2. Participants

Participants were adults aged ≥ 60 years who had not been diagnosed with dementia and were not receiving treatment for the condition. Furthermore, the participants were required to use a smartphone, understand and respond to the study questionnaire, understand the purpose of the study, and consent to participate. Exclusion criteria included a clinical diagnosis of dementia, severe sensory impairments (such as significant hearing or vision loss), major psychiatric disorders (e.g., major depressive disorder, schizophrenia), and serious medical illnesses that could interfere with participation. 

This study was approved from the Public Institutional Review Board (IRB No. P01-202504-01-053) and was registered with the Clinical Research Information Service (CRIS) (KCT0011586).

### 2.3. Interventions

#### Program Overview

The intervention in the study was conducted using MELI, a mobile application developed to prevent dementia. This application provided modules for cognitive training, physical activity, and mental-health promotion and encourages user participation through notification and feedback functions. On the main screen of the application, balanced recommendation activities (today’s activities) were presented every day and generally consisted of 2–3 activities such as cognitive games, emotion regulation activities, and exercise routines, which could be performed in an average of about 20 min. Participants were guided to use MELI 5 days a week; a study manager monitored the status of use and provided support if necessary. A detailed description of the application’s structure and content has been reported in a previous study [14].

**Figure 2:**
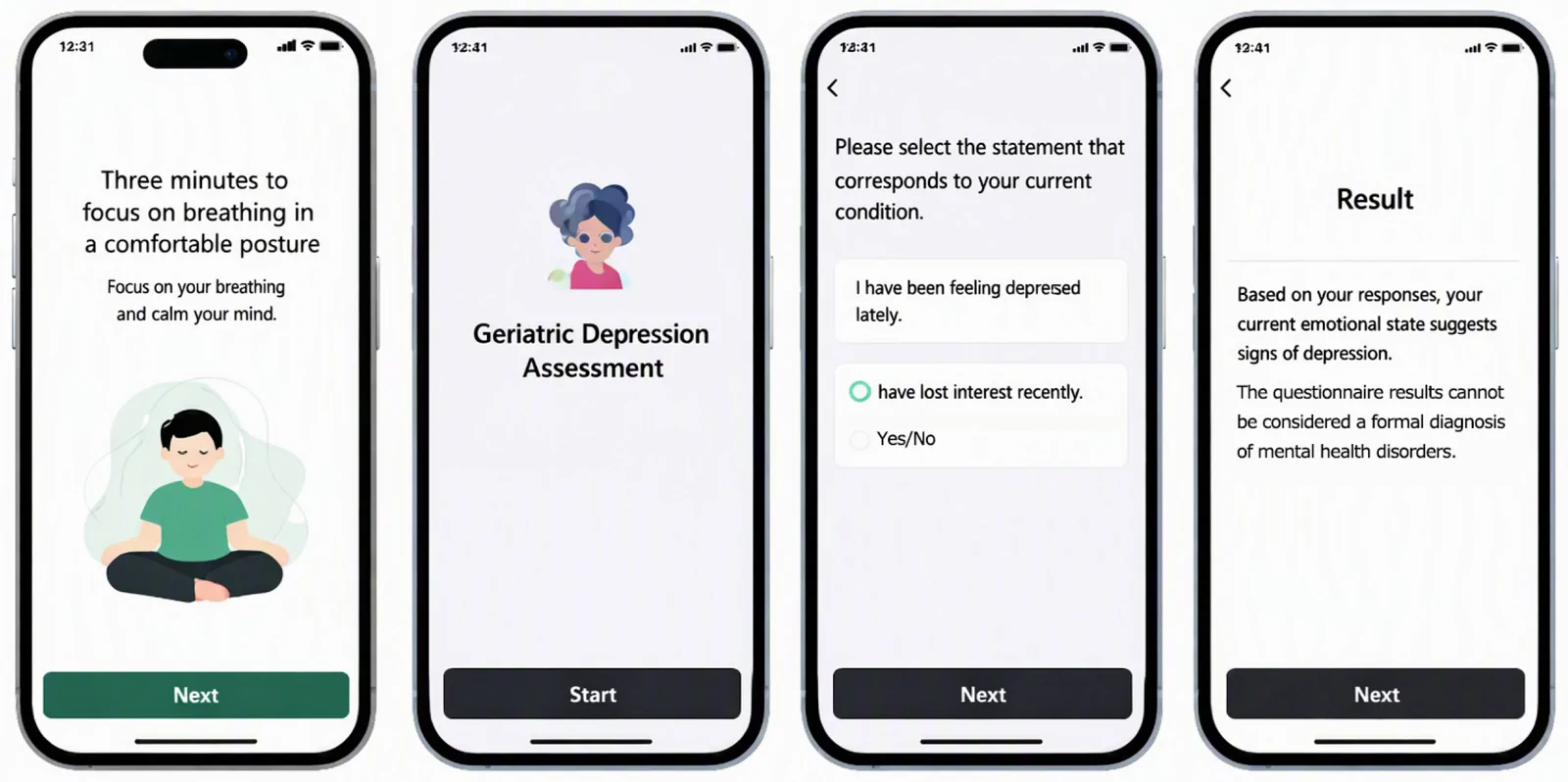
The MELI application.

The MELI application included modules for cognitive training, physical activity, and mental health promotion. The cognitive training module consisted of 15 games that aimed to improve memory, problem solving, language, attention, and space-time skills, some of which were developed based on standard neuropsychological tests such as the Trail Making Test and the Stroop Test; interval recall techniques were also applied. Physical-activity modules were designed to be easily performed in everyday life, including >90 types of exercise suggested by experts, and to help with cerebrovascular circulation and strengthening muscle strength. Mental-health promotion modules relieved tension and support emotional stability through relaxation activities such as breathing or butterfly hugs. In addition, participants could monitor their status by performing self-report questionnaires such as SMCQ, GDS, PSS, ISI-K and functional evaluations such as the TUG Test through the application. An example of the MELI application is presented in Figure 2.

### 2.4. Outcome Measures

#### 2.4.1. Assessment Schedule

The evaluation was conducted twice: before the dementia prevention application-based intervention and after the completion of the 7-month intervention period. The examination was conducted in person or by phone by the researcher in charge. There was no difference in evaluation methods between groups, and both methods followed the same standardized procedure. There was a possibility that the self-report results would be affected by the evaluation method, but the same guidelines were applied to minimize this measure. This study utilized standardized measurement tools whose reliability has been validated in Korea.

#### 2.4.2. Depression

Depression was assessed using the Korean version of the Short Geriatric Depression Scale (SGDS-K). The GDS is a self-administered tool developed to evaluate depressive symptoms in older adults [15]. In Korea, the GDS-K is used to suit the Korean cultural and linguistic context and consists of 30 items. The SGDS-K is a self-report tool that has been shortened to 15 items, and its reliability and validity have been verified in domestic studies [16]. Each item is scored using a yes/no response format, and total scores are calculated by summing individual item responses, with higher scores indicating more severe depressive symptoms. 

#### 2.4.3. Anxiety

Anxiety was assessed using the Korean version of the Hospital Anxiety and Depression Scale (HADS), a self-report tool developed to assess emotional states in hospital settings [17]. It has been translated into Korean, with its validity and reliability confirmed previously [18]. The HADS evaluates psychological distress through two main subscales: one for anxiety and another for depression. The HADS consists of two subscales that assess anxiety and depression separately. The HADS-A subscale used in this study comprises seven items, each rated on a 4-point Likert scale ranging from 0 to 3. Item scores were summed to generate a total anxiety score, with higher scores indicating greater levels of anxiety. 

#### 2.4.4. Life Satisfaction

To evaluate the life satisfaction, we used the Korean version of the Satisfaction with Life Scale (K-SWLS). The SWLS is a self-reported tool developed to assess overall life satisfaction [19]. The Korean version was translated, and its reliability and validity were verified [20]. The K-SWLS comprises five items, each rated on a 7-point Likert scale ranging from 1 to 7. Total scores were calculated by summing the responses to all items, with higher scores indicating greater life satisfaction.

### 2.5. Sample Size Calculation

To estimate the required sample size, G*Power (version 3.1) for a repeated measures design was used. Assuming an large effect size of 0.40, a significance level (α) of 0.05, and a statistical power of 0.80, a minimum of 24 participants per group was required. Considering the possibility of dropping out, 58 participants were finally recruited. Twelve participants dropped out, resulting in a final analysis of 46 participants (23 per group).

### 2.6. Statistical Analysis

Data analysis was performed using SPSS (version 22.0, IBM Corp., Armonk, NY, USA). A homogeneity test was conducted to analyze demographics of the study participants. Prior to the main analysis, model assumptions were examined. Levene’s test confirmed homogeneity of variances, whereas normality tests (Kolmogorov–Smirnov and Shapiro–Wilk) indicated that some variables did not fully meet the assumption of normality. For the main analysis, repeated-measures analysis of covariance (ANCOVA) was performed to examine group differences over time while controlling for baseline values. 

For all statistical analyses, statistical significance was defined as *p* < 0.05. Effect sizes were calculated using partial eta squared (η^2^). 

## 3. Results

Baseline characteristics and homogeneity test results of the participants are presented in Table 1. The experimental and control groups had a mean age of 72.35 years and 68.96 years, respectively, indicating a slightly higher mean age in the experimental group. Thus, a statistically significant difference in age was observed between the two groups (*p* < 0.05).

The sex distribution was as follows: 2 males (8.7%) and 21 females (91.3%) in the experimental group, and 4 males (17.4%) and 19 females (82.6%) in the control group. No significant differences were observed between the groups at baseline (*p* > 0.05). Baseline K-SGDS and K-SWLS scores also did not differ significantly between the groups (*p* > 0.05). However, at baseline, significant differences were found in K-CIST and K-HADS-A scores between the two groups.

After controlling for age, a repeated-measures ANCOVA was conducted with group (intervention vs. control) as a between-subjects factor and time (pretest vs. posttest) as a within-subjects factor. The analysis revealed a significant group × time interaction for the K-SGDS (F(1, 35) = 10.28, *p* = 0.003, partial η^2^ = 0.227). Adjusted means indicated that the depression score of the intervention group decreased from 2.07 (SE = 0.86, 95% CI [0.33, 3.81]) to 1.59 (SE = 0.76, 95% CI [0.04, 3.14]), whereas that of the control group increased from 2.65 (SE = 0.69, 95% CI [1.25, 4.05]) to 3.44 (SE = 0.61, 95% CI [2.20, 4.68]).

**Figure 3:**
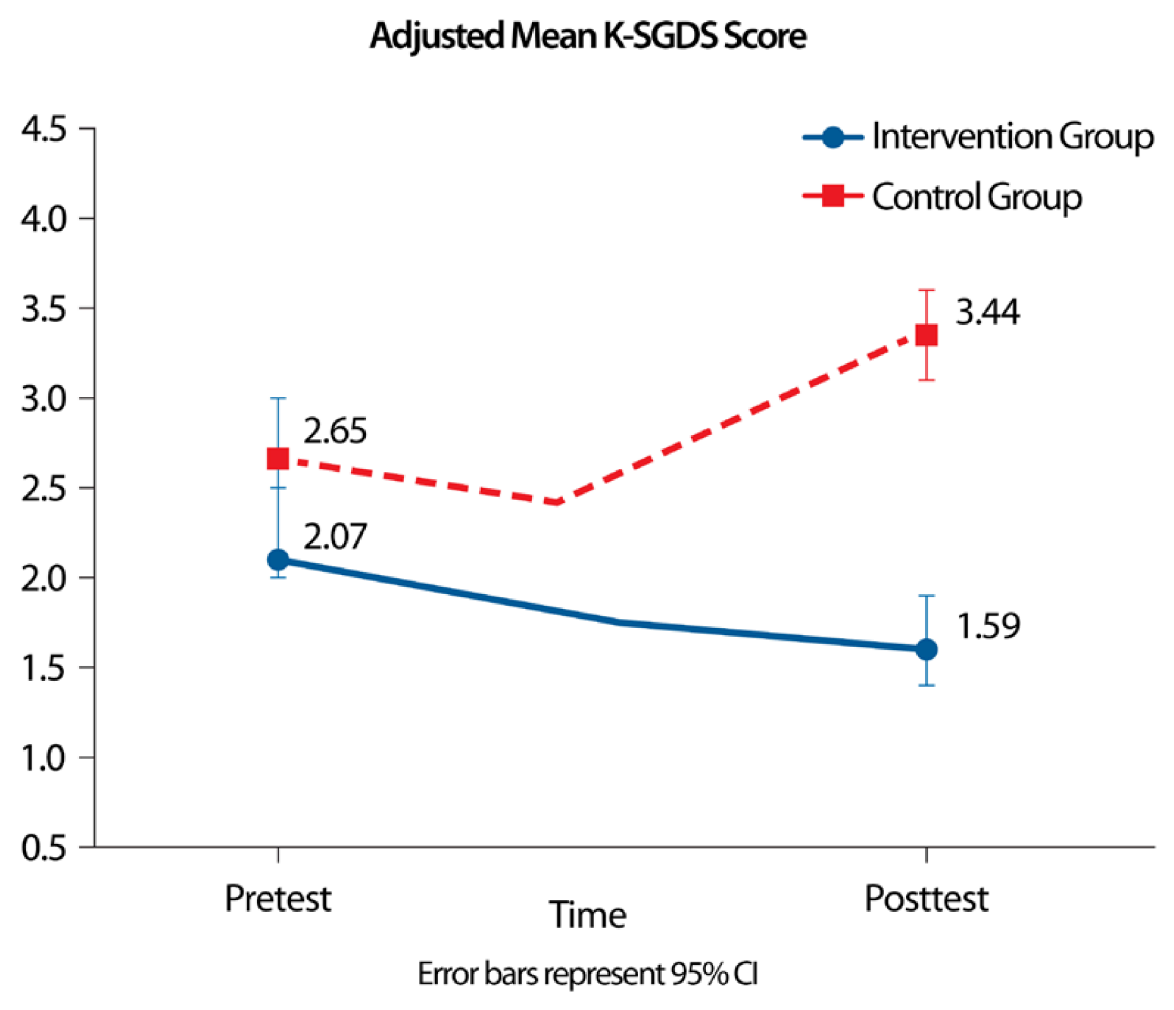
Changes in K-SGDS Scores.

In contrast, the group × time interaction for the K-SWLS was not significant (F(1, 43) = 0.01, *p* = 0.933, partial η^2^ = 0.000), indicating no differential change in life satisfaction between groups. The difference in degrees of freedom between the two outcomes reflects the existence of missing data on the K-SGDS measure, which reduced the analytic sample, while the full sample was available for the K-SWLS analysis. Table 2 summarizes these repeated-measures ANCOVA results. Figure 3 illustrates changes in adjusted mean depression scores (K-SGDS) between the intervention and control groups.

After controlling for age and baseline anxiety, the adjusted mean posttest anxiety score was significantly lower in the intervention group (M = 2.10, SE = 0.34, 95% CI [1.41, 2.79]) than in the control group (M = 3.25, SE = 0.34, 95% CI [2.56, 3.94]) (F(1, 42) = 5.21, *p* = 0.028, partial η^2^ = 0.110) (Table 3).

## 4. Discussion

This study investigated effects of application-based dementia prevention intervention on depressive symptoms and life satisfaction in the elderly living in local communities in Korea. In the basic analysis, the experimental and control groups were similar in most demographic and psychological characteristics, but differences were observed in the K-CIST and K-HADS-A scores. To consider these baseline differences, age and prior score were included as covariates in the subsequent analysis. As a result of repeated-measures ANCOVA controlling for baseline differences, the interaction effect between group and time was significant. The intervention group displayed greater improvements in depression and anxiety levels than the control group. These findings suggest that application-based dementia prevention interventions have a positive impact on emotional health (reducing depression and anxiety).

Previous application-based dementia prevention studies have primarily focused on enhancing cognitive function and reducing dementia risk factors. More recent dementia prevention research has emphasized multidomain interventions that comprehensively address cognition, physical activity, and lifestyle habits [21]. Qiu et al. investigated the effectiveness of a mobile-based multidomain intervention, setting cognitive function as the primary outcome variable and assessing depression, quality of life, and other secondary measures [22]. Although their study reported significant improvements in cognitive function and quality of life, no statistically significant change was noted in depression. In contrast, in the present study, we observed improvements in depression and anxiety, although no significant change in life satisfaction (K-SWLS). Although both studies assessed depression, their results differed, which might be explained by differences in participant characteristics, timing of assessment, and intervention methods. Furthermore, while the present study measured life satisfaction, the previous study measured quality of life. Although these instruments are not identical, they address related constructs and were therefore compared. The differing results of these studies indicate that life satisfaction, as a broader and more subjective construct encompassing multiple sub-domains, may be less sensitive to short-term intervention effects. In addition, although not a dementia prevention study, the meta-analysis conducted by Linardon et al. on smartphone-mediated mental health strategies is another relevant line of research. That study reported statistically significant reductions in depression and anxiety symptoms, despite the small overall effect size [9], which were consistent with the findings of the present study although its small effect size also warrants cautious interpretation. Taken together, these results indicate that application-based health management can be usefully applied to emotional-health management. In the same context, application-based interventions for dementia prevention may also have the potential to contribute to mental-health promotion.

This study has some limitations. First, the research period was relatively short in terms of research design, making it difficult to verify the long-term effects of the application and to closely analyze the relationship between the intervention effects and the actual program use pattern owing to the lack of objective data on application adherence. Second, a limitation of the measurement method is that psychological indicators such as depression and life satisfaction were measured through a self-report questionnaire, and the comprehensive analysis was limited because cognitive scores could not be measured after using the application. Third, there was a base difference in K-CIST and K-HADS-A between the intervention and control groups in the statistical analyses, and the baseline value was included as a covariate to minimize this limitation, but care must be taken when interpreting the results because these adjustments may not completely eliminate the effect. Only some variables satisfied normality, possibly affecting the assumptions of the statistical test; thus, the results should be interpreted with caution, and a larger sample size or nonparametric tests should be employed in future studies. Finally, regarding the effect of study participation, the possibility that the Hawthorne effect, which can change behavior or emotional response, influenced the results could not be completed ruled out. Follow-up studies are needed to review the effectiveness of digital-based dementia prevention interventions more systematically and reliably by addressing the aforementioned limitations.

In this study, the term “dementia prevention” reflects the design purpose of the MELI application, intended to lower the risk of dementia through cognitive training, physical activity, and emotional support. However, since the actual onset of dementia was not directly measured, these findings should be interpreted as improvements in emotional health that may contribute to reducing dementia risk, rather than as strong evidence of dementia prevention itself. Furthermore, because dementia includes multiple subtypes, such as Alzheimer’s disease, vascular dementia, frontotemporal dementia, Lewy body dementia, and Parkinson’s-related dementia, it could not be strictly defined as the prevention of all forms.

Nonetheless, this study is important as it examined the effects of a mobile application-based dementia prevention intervention on emotional outcomes in older adults. Unlike previous studies that primarily focused on cognitive function enhancement, this study assessed key psychological outcomes, including anxiety, depression and life satisfaction, thereby providing a more multidimensional verification of the effects of digital dementia prevention interventions. These results suggest that mobile application-based interventions can be utilized not only to improve cognitive health, but also to manage emotional health in older adults, highlighting the potential for digitally assisted interventions in future community-based dementia prevention strategies.

## 5. Conclusions

The findings of this study indicate that MELI-based dementia prevention application might contribute to positive changes in the emotional health of older adults. Our analysis indicated improvements in depression and anxiety scores, whereas changes in life satisfaction did not reach statistical significance. Therefore, digital dementia prevention interventions retain the potential to support emotional well-being. Nonetheless, the hereby-presented results were not consistent across all measures and should thus be interpreted with caution. Nevertheless, this study is meaningful in that it adopted a multidimensional approach by including psychological indicators (e.g., depression, anxiety, and life satisfaction) rather than focusing solely on cognitive outcomes as in previous research. Future studies should address the limitations of the present work and examine the effects of digital dementia prevention interventions, with particular attention to the nuanced aspects of emotional health.

## Figures and Tables

**Table 1:** Baseline characteristics of participants.

Variable	Intervention (n = 23)	Control (n = 23)	Test	*p*-Value
Age			t	
Mean ± SD	72.35 ± 3.74	68.96 ± 6.99		0.048 *
Sex, (%)			Chi-square	0.381
Male	2 (8.7)	4 (17.4)		-
Female	21 (91.3)	19 (82.6)		-
K-CIST (baseline)	28.00 ± 2.43	25.52 ± 3.75	t	0.011 *
K-SGDS (baseline)	2.20 ± 3.95	2.57 ± 2.74	t	0.738
K-HADS-A (baseline)	4.13 ± 2.72	2.57 ± 2.11	t	0.034 *
K-SWLS (baseline)	18.08 ± 4.31	19.17 ± 3.62	t	0.360

* *p* < 0.05. Values are presented as mean ± standard deviation (SD) or number (%). t, independent *t*-test; Chi-square, chi-square test. K-CIST, Korean version of the Cognitive Impairment Screening Test. K-SGDS, Korean version of the Short Geriatric Depression Scale. K-HADS-A, Korean version of the Hospital Anxiety and Depression Scale-Anxiety Subscale. K-SWLS, Korean version of the Satisfaction with Life Scale.

**Table 2:** Repeated-measures analysis of covariance results for K-SGDS and K-SWLS (covariate: age).

Outcome	Group	Pre Adjusted M ± SE, 95% CI	Post Adjusted M ± SE 95% CI	Effect	F	*p*-Value	Partial η^2^
K-SGDS	Intervention	2.07 ± 0.86 [0.33, 3.81]	1.59 ± 0.76 [0.04, 3.14]	Group × Time	10.28	0.003 *	0.227
	Control	2.65 ± 0.69 [1.25, 4.05]	3.44 ± 0.61 [2.20, 4.68]				
K-SWLS	Intervention	18.18 ± 0.86 [16.44, 19.91]	18.58 ± 0.71 [17.15, 20.02]	Group × Time	0.01	0.933	0.000
	Control	19.09 ± 0.86 [17.36, 20.82]	19.42 ± 0.71 [17.99, 20.85]				

* *p* < 0.05. M, mean; η^2^, partial eta squared. K-SGDS, Korean version of the Short Geriatric Depression Scale. K-SWLS, Korean version of the Satisfaction with Life Scale.

**Table 3:** Baseline-adjusted analysis of covariance results for K-HADS-A.

Group	Adjusted Mean	SE	95% CI	F	*p*-Value	Partial η^2^
Intervention	2.10	0.34	[1.41, 2.79]	5.21	0.028 *	0.110
Control	3.25	0.34	[2.56, 3.94]			

* *p* < 0.05. M, mean;.η^2^, partial eta squared.

## Data Availability

The datasets analyzed during the current study are publicly available (https://doi.org/10.6084/m9.figshare.32671722).

## Abbreviations

ANCOVA	Analysis of Covariance
CRIS	Clinical Research Information Service
G*Power	Statistical Power Analysis Program
GDS	Geriatric Depression Scale
HADS	Hospital Anxiety and Depression Scale
HADS-A	Hospital Anxiety and Depression Scale–Anxiety Subscale
IRB	Institutional Review Board
IT	Information Technology
K-CIST	Korean version of the Cognitive Impairment Screening Test
K-HADS-A	Korean version of the Hospital Anxiety and Depression Scale–Anxiety Subscale
K-SGDS	Korean version of the Short Geriatric Depression Scale
K-SWLS	Korean version of the Satisfaction with Life Scale
mHealth	Mobile Health
MELI	Dementia prevention mobile application MELI
SD	Standard Deviation
SGDS	Short Geriatric Depression Scale
SGDS-K	Korean version of the Short Geriatric Depression Scale
SPSS	Statistical Package for the Social Sciences
SWLS	Satisfaction with Life Scale
η^2^	Partial Eta Squared

## References

[1] Moll van Charante E.P., Hoevenaar-Blom M.P., Song M., Andrieu S., Barnes L., Birck C., Brooks R., Coley N., Eggink E., Georges J. (2024). PRODEMOS study group. Prevention of dementia using mobile phone applications (PRODEMOS): A multinational, randomized, controlled effectiveness–implementation trial. Lancet Healthy Longev..

[2] GBD 2019 Dementia Forecasting Collaborators (2022). Estimation of the global prevalence of dementia in 2019 and forecasted prevalence in 2050: An analysis for the Global Burden of Disease Study 2019. Lancet Public Health.

[3] Suvish, Ghamari M., Sundaram S. (2025). Digital Dementia: Smart Technologies, mHealth Applications and IoT Devices, for Dementia-Friendly Environments. J. Sens. Actuator Netw..

[4] Zhang X., Guo T., Zhang Y., Jiao M., Ji L., Dong Z., Li H., Chen S., Zheng W., Jing Q. (2024). Global burden of Alzheimer’s disease and other dementias attributed to metabolic risks from 1990 to 2021: Results from the Global Burden of Disease Study 2021. BMC Psychiatry.

[5] Shah H.A., Khalil S., Andberg S., Koivisto A.M., Bednarik R. (2025). Eye tracking based detection of mild cognitive impairment: A review. Inf. Fusion.

[6] Wang Y., Liu S., Spiteri A.G., Huynh A.L.H., Chu C., Masters C.L., Goudey B., Pan Y., Jin L. (2024). Understanding machine learning applications in dementia research and clinical practice: A review for biomedical scientists and clinicians. Alzheimer’s Res. Ther..

[7] Elser H., Horváth-Puhó E., Gradus J.L., Smith M.L., Lash T.L., Glymour M.M., Sørensen H.T., Henderson V.W. (2023). Association of Early-, Middle-, and Late-Life Depression With Incident Dementia in a Danish Cohort. JAMA Neurol..

[8] Xiao X., Li Y., Wu Q., Liu X., Cao X., Li M., Liu J., Gong L., Dai X.-J. (2025). Development and validation of a novel predictive model for dementia risk in middle-aged and elderly depression individuals: A large and longitudinal machine learning cohort study. Alzheimer’s Res. Ther..

[9] Linardon J., Cuijpers P., Carlbring P., Messer M., Fuller-Tyszkiewicz M. (2019). The efficacy of app-supported smartphone interventions for mental health problems: A meta-analysis of randomized controlled trials. World Psychiatry.

[10] Salahub C., Austin P.C., Bai L., Ivers N.M., Jones A., Tadrous M., Tran J., Lapointe-Shaw L. (2025). Days at home for older adults receiving a remote monitoring intervention compared with usual home care recipients. J. Am. Med. Dir. Assoc..

[11] Cho E., Yang M., Kim M.J., Hwang S., Kim E., Cho J. (2024). Effectiveness of a mobile app-based individualized non-pharmacological intervention on behavioral and psychological symptoms of dementia in community-dwelling older adults: Study protocol for a randomized control trial. J. Korean Gerontol. Nurs..

[12] Han J.W., Oh D.J., Kim T.H., Kwak K.P., Kim B.J., Kim S.G., Kim J.L., Moon S.W., Park J.H., Ryu S.-H. (2025). Refining Western dementia-risk paradigms: Evidence from a decade of the Korean longitudinal study on cognitive aging and dementia. J. Korean Med. Sci..

[13] Qiu Y., Wu M., Liu J., Li C., Yu Y., Zeng L., Yang F., Zhang X., Chen G. (2025). Effectiveness of information technology-based cognitive behavioral therapy on depression and anxiety symptoms among older adults: Systematic review and meta-analysis. Gen. Hosp. Psychiatry.

[14] Han S.H., Kong S.H., Kim N.H., Min K.B. (2025). The effectiveness of the comprehensive dementia prevention app ‘MELI’. Asia-Pac. J. Converg. Res. Interchange.

[15] Yesavage J.A., Brink T.L., Rose T.L., Lum O., Huang V., Adey M., Leirer V.O. (1983). Development and validation of a geriatric depression screening scale: A preliminary report. J. Psychiatr. Res..

[16] Bae J.N., Cho M.J. (2004). Development of the Korean version of the Geriatric Depression Scale and its short form among elderly psychiatric patients. J. Psychosom. Res..

[17] Zigmond A.S., Snaith R.P. (1983). The hospital anxiety and depression scale. Acta Psychiatr. Scand..

[18] Se-Man O.H., Min K.J., Park D.B. (1999). A study on the standardization of the hospital anxiety and depression scale for Koreans: A comparison of normal, depressed and anxious groups. J. Korean Neuropsychiatr. Assoc..

[19] Diener E., Emmons R.A., Larsen R.J., Griffin S. (1985). The Satisfaction With Life Scale. J. Pers. Assess..

[20] Lim Y.J. (2012). Psychometric Properties of the Satisfaction with Life Scale among Korean Police Officers, University Students, and Adolescents. Korean J. Psychol. General..

[21] Kivipelto M., Mangialasche F., Ngandu T. (2018). Lifestyle interventions to prevent cognitive impairment, dementia and Alzheimer disease. Nat. Rev. Neurol..

[22] Qiu Y.F., Wu M., Liu J.L., Li C.Y., Yu Y.Q., Zeng L.J., Yang B.X., Yang F. (2024). Effectiveness of digital intelligence interventions on depression and anxiety in older adults: A systematic review and meta-analysis. Psychiatry Res..

